# Hierarchical Template Matching for 3D Myocardial Tracking and Cardiac Strain Estimation

**DOI:** 10.1038/s41598-019-48927-2

**Published:** 2019-08-28

**Authors:** Jayendra M. Bhalodiya, Arnab Palit, Enzo Ferrante, Manoj K. Tiwari, Sunil K. Bhudia, Theodoros N. Arvanitis, Mark A. Williams

**Affiliations:** 1 Warwick Manufacturing Group (WMG), University of Warwick, CV4 7AL Coventry, United Kingdom; 2 Instituto de Investigación en Señales, Sistemas e Inteligencia Computacional, sinc(i), FICH-UNL/CONICET, Santa Fe, Argentina; 3 Indian Institute of Technology Kharagpur, 721302 Kharagpur, West Bengal India; 4 Royal Brompton and Harefield NHS Foundation Trust, SW3 6NP, London, United Kingdom; 5 Institute of Digital Healthcare, WMG, University of Warwick, CV4 7AL Coventry, United Kingdom

**Keywords:** Biomedical engineering, Computer science

## Abstract

Myocardial tracking and strain estimation can non-invasively assess cardiac functioning using subject-specific MRI. As the left-ventricle does not have a uniform shape and functioning from base to apex, the development of 3D MRI has provided opportunities for simultaneous 3D tracking, and 3D strain estimation. We have extended a Local Weighted Mean (LWM) transformation function for 3D, and incorporated in a Hierarchical Template Matching model to solve 3D myocardial tracking and strain estimation problem. The LWM does not need to solve a large system of equations, provides smooth displacement of myocardial points, and adapt local geometric differences in images. Hence, 3D myocardial tracking can be performed with 1.49 mm median error, and without large error outliers. The maximum error of tracking is up to 24% reduced compared to benchmark methods. Moreover, the estimated strain can be insightful to improve 3D imaging protocols, and the computer code of LWM could also be useful for geo-spatial and manufacturing image analysis researchers.

## Introduction

Cardiovascular Disease (CVD) is an important burden on the global population. It accounts for almost 31% of the mortality^1^. CVD covers a spectrum of diseases but mainly dysfunction of the heart muscles, from various aetiologies, and heart rhythm. The heart can be investigated and assessed using invasive and increasingly more non-invasive techniques^2^. Magnetic resonance imaging (MRI) with 3D imaging capabilities is gaining more support and applications^2,3^. The assessment of myocardium (heart wall muscles) provides clinical experts with the details of CVD diagnosis, prognosis and therapeutic interventions^2,4,5^. The details such as left ventricular (LV) myocardial tracking and strain (shortening or lengthening of the myocardium muscles) could objectively quantify myocardial health, providing a better understanding of cardiac diseases such as cardiomyopathy, myocardial infarction, arrhythmia, and valvular diseases^2,6^.

Heart muscles shrink, expand, rotate and create torsion simultaneously during a cardiac cycle and a common complication that arises in left ventricular (LV) myocardial tracking is the consideration of longitudinal heart movement. As a conventional or common practice, multiple 2D short-axis and 2D long-axis images are used to construct 3D myocardial motion and strain^7–9^. However, the approach is prone to 2D slice misregistration, patient cooperation, and dependent on radiographer (subjective)^10^, because the acquisition of 2D long-axis and 2D short-axis images happen at a different time while scanning a patient. As a result, 3D tagging is emerged as a promising method to allow reconstruction of 3D myocardial strain from single image volume rather than multiple different 2D images^10–12^. However, methods such as HARmonic Phase (HARP), Strain Encoding (SENC) are natively 1D or 2D, and merged with other methods or multiple 2D long- and short-axis images to calculate 3D myocardial strain^11,13,14^. However, a limited number of long-axis images could affect the accuracy of such tracking^11^. This fact motivates the use of 3D MRI instead of 2D short- and long-axis images, making it crucial to perform accurate 3D myocardial tracking. Medical image analysis researchers have contributed algorithms using direct detection-based methods^15^, Fourier-based methods^13,14,16^, tracking-based methods^17–22^, and block-matching-based methods^17,23–25^. Direct detection-based methods detect and track tag intersection points which could avoid image artefacts but too sparse tag points could limit the accuracy of myocardial tracking^12,26^. Fourier-based methods (for example HARP^14^) exploit the correspondence property of a local shift in the spatial domain and a phase shift in the Fourier domain but the method is limited to use tagged MRI^12^. Tracking-based methods optimize similarity between frame intensities and estimates transformation model, however, they can be dependent on a spatial regularization which could limit the accuracy^12^.

The block-matching approach has provided promising results using 3D ultrasound images^17^, and 2D MRI^25^. The standard myocardial tracking technique uses 3D speckle tracking through ultrasound images^2,17^. The method tracks an inherent pattern (speckle) of ultrasound imaging using a matching kernel (block or volume-of-interest), and the sum of absolute difference as a block-matching criterion. However, the accuracy of such a method is highly dependent on the size of the kernel, as too large or too small kernels could lead to incorrect tracking^17^. Such technical limitation could be overcome by using a hierarchy of matchings, which can provide a promising correlation up to the smallest level of the matching^25,27^. The hierarchical matching using 2D MRI is reported with promising accuracy in the literature^25^, however, 3D myocardial tracking using 3D MRI has received less attention in the literature^12^. Therefore, in this article, we have extended the standard block-matching technique by incorporating a hierarchical matching for 3D myocardial tracking.

Myocardial tracking algorithms use a transformation function to establish a point-to-point correspondence between two images^28^. Spline-based functions are commonly used as they can efficiently handle image deformations^11,20,29,30^. However, researchers have reported that the spline functions can generate large errors when the spacing between points is irregular^28^. It is also reported that the Cartesian coordinate B-spline model can generate errors due to ill-conditioned and ill-posed polynomials^30^. To address this issue, other approaches are suggested such as cylindrical coordinate B-spline model^30^ (as LV is roughly cylindrical), explicit regularization^29^ and local weighted-mean transformation function^25,28,31,32^. The cylindrical coordinate system and regularisation function with B-spline function could lead to detailed and complicated design and planning of myocardial tracking, whereas, Local Weighted Mean (LWM) function is proposed in the literature to overcome the elaborated procedure of spline interpolation^33^. The function is reported advantageous over the thin-plate spline and multi quadratics as the LWM function does not need a solution of a very large system of equations^28^. LWM functions can provide up to sub-pixel smoothness and accuracy in the displacement gradient^28^. Moreover, the averaging procedure of the LWM smoothes the noise in correspondences^28^. The function is adapted by researchers for 2D image registration of geo-spatial images^28,32,34^ and reviewed for medical imaging^31^. Previously, we show the promising results of 2D LWM function for 2D cardiac image registration^25^. In this work, we propose an extension of the standard 2D LWM function to 3D and use it to perform 3D myocardial tracking.

In this article, we extend a Hierarchical Template Matching (HTM)^25^ method for 3D myocardial tracking and strain estimation. The 3D LWM function is introduced and adapted for 3D myocardial tracking pipeline. The objective is to improve numerical stability in myocardial tracking by reducing maximum error and outliers. The 3D myocardial tracking and strain calculation is performed using state-of-the-art tagged MRI dataset^12^ of 15 healthy patients. The method is validated using four different strategies which include tracking of known LV points, calculating displacement, strain values and eigenvalues analysis in a cardiac cycle.

## Methods and Material

### Proposed method

The objective of the proposed method is to track 3D myocardial points, and calculate strain values. The overall flow of the method is divided into three steps: (i) Segmentation of 3D volumes of LV, (ii) Hierarchical block-matching and calculation of LWM function, (iii) Myocardial point tracking and strain calculation. The Biomedical and Scientific Research Ethics Committee (BSREC) approval to conduct the research is obtained from the University of Warwick (REGO-2016-1865). The study involves anonymised data of healthy volunteers which are downloaded from publicly available data repository^12^. The data was previously collected, anonymized, and made publicly available by the authors of the literature^12^. We have not recruited any participants for our study. We have developed and tested our algorithm using this anonymised and publicly available dataset.

#### Segmentation of 3D volume of LV

**Figure 1:**
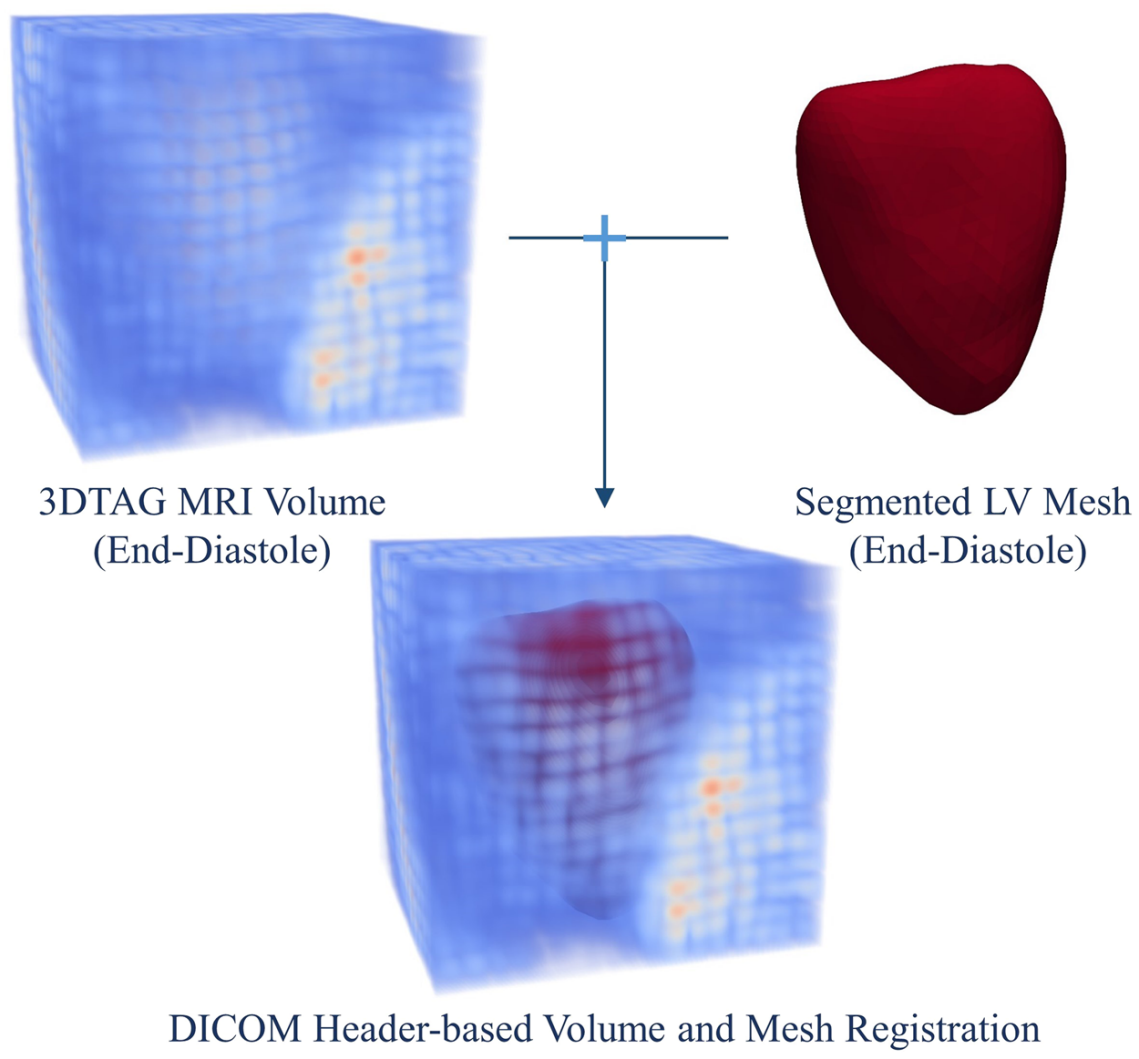
The 3DTag MRI volume, and segmented LV mesh.

The entire cardiac cycle is covered with multiple 3D MRI volumes. These volumes are collected as mentioned in the dataset section (see Section 1.2.1). The segmented LV for each patient is also collected from the same dataset. In each patient, the segmentation of end-diastolic LV volume is collected. The segmented LV is obtained from steady-state free precision images at end-diastole (as mentioned by dataset providers^12^). As shown in Fig. 1, the registration of segmented LV mesh and MRI volume at end-diastole is performed using the dicom file header information as per dataset guidelines. The registered LV mesh is tracked in all the frames of a cardiac cycle for myocardial tracking.

#### Hierarchical matching and calculation of LWM function

**Figure 2:**
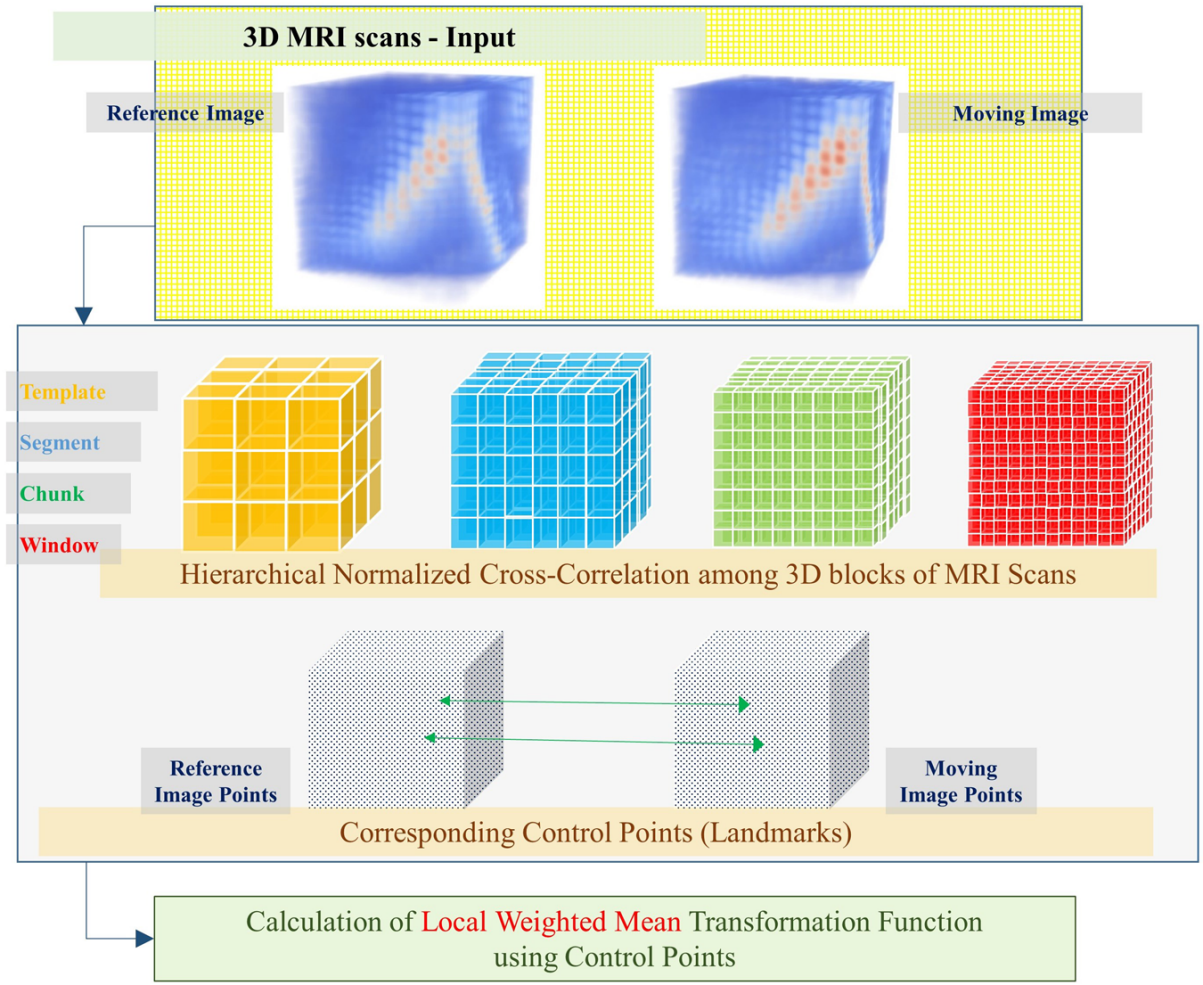
The overall flow of hierarchical matching and transformation function calculation.

Figure 2 depicts the proposed hierarchical matching approach for a pair of reference and moving images. The reference image is the end-diastolic image, while the moving image is a current frame of the cardiac cycle. The method is inspired by existing box-matching-based tracking^17^ and landmark-based non-rigid image registration^28,31^ algorithms. We propose a three steps method with the following stages: (a) Generating control points in moving image that will be interpreted as landmarks, (b) matching corresponding control points in the reference image, and (c) calculating a dense transformation function from the sparsely matched landmarks.

**Figure 3:**
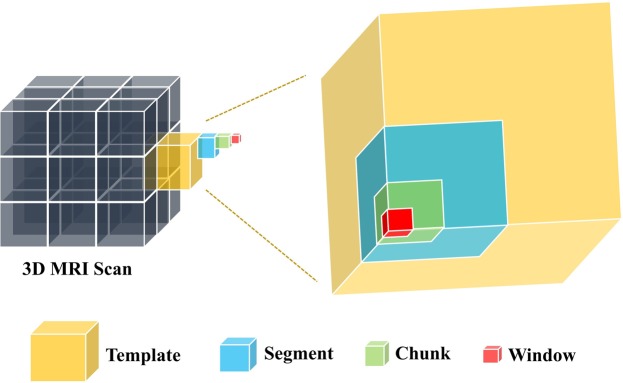
A pictorial representation of the different sized image blocks (Template, Segment, Chunk, Window).

Generating control points in moving image: We generate a set of regular moving image points (also referred in this work as landmarks or control points) by dividing the moving image into multiple blocks of size t × t × t, which are defined as templates (see Fig. 3). Each template is further divided into segments of size t/2 × t/2 × t/2. Each segment is divided into t/4 × t/4 × t/4 size chunks, and each chunk is divided into t/8 × t/8 × t/8 size windows. It should be noted that throughout this article we will use the word ‘block’ to describe template, segment, chunk or window interchangeably. We define the first point of each window as its representative point (or landmark). The set of all representative points in the moving image is therefore defined as ${P}_{moving}=\{{m}_{1},{m}_{2},\ldots ,{m}_{n}|n=total\,number\,of\,Windows\}$.

The template size t × t × t pixels is determined considering literature^3,17,25^ and small in-house analysis. Previous work on speckle tracking has mentioned that template size 8 could perform myocardial tracking in ultrasound images^17^. The MRI feature tracking researchers have mentioned that the larger template size is required to capture large muscular deformations^3^, and a hierarchy of templates could improve matching^25^. Therefore, we have selected template size as 16 × 16 × 16 pixels with a hierarchy of segments of 8 × 8 × 8 pixels, chunks of 4 × 4 × 4 pixels, and windows of 2 × 2 × 2 pixels.

Matching corresponding control points in the reference image: The matching of moving image landmarks is performed using 3D Normalized Cross-Correlation (NCC)^27,35,36^. As mentioned in Equation (1), the NCC returns the value of a correlation coefficient (CC) at each point of the image, which ranges from -1.0 to + 1.0. The maximum value of the CC matrix leads to the matching location. The correlation coefficient λ is defined in Equation (1).

1 $$\lambda =\frac{{\sum }^{}[f(x,y,z)-{\bar{f}}_{p,q,r}][b(x-p,y-q,z-r)-\bar{b}]}{\sqrt{{\sum }^{}{[f(x,y,z)-{\bar{f}}_{p,q,r}]}^{2}{\sum }^{}{[b(x-p,y-q,z-r)-\bar{b}]}^{2}}}$$

In Equation (1), *b* represents 3D moving image block and $\overline{b}$ is the mean intensity value, *f* represents the reference image and $\overline{f}$ is the mean intensity of reference image which is covered under the block *b*.

**Figure 4:**
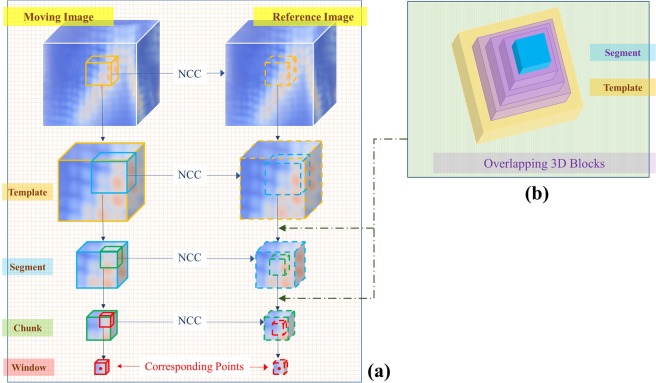
A pictorial representation of the workflow of the proposed method. **(a)** The hierarchical matching process **(b)** pyramid structure of overlapping blocks, which are inserted between the Template and Segment layers, and between the segment and chunk layers.

Each template of the moving image is slid over the reference image, and CC at each location of the reference image is calculated using Equation (1). The maximum value of CC leads to the location of the matching template of the reference image. After that, each moving template is divided into four segments. Each segment is slid over the reference template, and CC is calculated. The maximum CC value leads to the location of the corresponding segment of the reference image. Similarly, each moving segment is divided into four chunks. Each moving chunk is matched with reference image chunk using CC. Finally, each moving chunk is divided into four windows. Each moving window is matched with reference image window using CC. The pictorial representation of the process is shown in Fig. 4. The matching procedure gives the correspondence between a moving image block, and a reference image block. The set of first points of each reference image window creates a reference point set. ${P}_{reference}=\{{r}_{1},{r}_{2},\ldots ,{r}_{n}|n=total\,number\,of\,Windows\}$.

The matching process discussed in the previous paragraph could result in incorrect point correspondences. To update these incorrect point correspondences, the pyramidal approach is used as overlapping blocks. An overlapping block is defined as a surrounding block of a Segment. Each Segment is surrounded by three overlapped blocks of different sizes, creating a pyramid structure. If a Segment and its overlapped blocks match in the same hierarchy within the corresponding Template then the matching Segment is considered as a true matching Segment. Similarly, each Chunk is surrounded by an overlapped block. If a Chunk and its overlapped block match in the same hierarchy within the corresponding Segment then the matching Chunk is considered as a true match. If the match is not a true match then the point is set as a non-displaced point.

Calculating Transformation Function – LWM: A radial basis local weighted mean (LWM) function calculates transformation between a moving image and a reference image using a set of control points (as derived in section 1.1.2.a and 1.1.2.b). The calculated LWM function is used to map every point from the moving image to the reference image. It is desired to estimate a dense LWM transformation function between a moving image and a reference image, given that the sparse correspondences between the moving and reference image are known. We formulate the problem as follows.

As a first step, the sets of corresponding matched control points of the moving and reference image $\{({x}_{i},{y}_{i},{z}_{i}),\,({X}_{i},{Y}_{i},{Z}_{i}):i=1,\ldots ,N\}$, are organized as $\{({x}_{i},{y}_{i},{z}_{i},{X}_{i}):i=1,\ldots ,N\}$, $\{({x}_{i},{y}_{i},{z}_{i},{Y}_{i}):i=1,\ldots ,N\}$, $\{({x}_{i},{y}_{i},{z}_{i},{Z}_{i}):i=1,\ldots ,N\}$. After that, we determine polynomials $Pol{y}_{i,x}$, $Pol{y}_{i,y}$ and $Pol{y}_{i,z}$ that fit *i*^*th*^ control point and its (*n* − 1) nearest neighbour control points. The polynomials are second order polynomials with ten coefficients. $Pol{y}_{i,x}$ is used to calculate X-component of the transformed point, $Pol{y}_{i,y}$ is used to calculate Y-component of the transformed point, and $Pol{y}_{i,z}$ is used to calculate Z-component of the transformed point.

A random point *p* = (*x*, *y*, *z*) can be transformed using the weighted mean of all polynomials passing over *p*. The transformed X-component, Y-component and Z-component of a transformed point can be calculated as defined in Equation (2).

2 $$\begin{array}{c}X(x,y,z)=\frac{{\sum }_{i=1}^{N}W\{{[{(x-{x}_{i})}^{2}+{(y-{y}_{i})}^{2}+{(z-{z}_{i})}^{2}]}^{\frac{1}{2}}/{R}_{n}\}Pol{y}_{i,x}(x,y,z)}{{\sum }_{i=1}^{N}W\{{[{(x-{x}_{i})}^{2}+{(y-{y}_{i})}^{2}+{(z-{z}_{i})}^{2}]}^{\frac{1}{2}}/{R}_{n}\}}\,,\\ Y(x,y,z)=\frac{{\sum }_{i=1}^{N}W\{{[{(x-{x}_{i})}^{2}+{(y-{y}_{i})}^{2}+{(z-{z}_{i})}^{2}]}^{\frac{1}{2}}/{R}_{n}\}Pol{y}_{i,y}(x,y,z)}{{\sum }_{i=1}^{N}W\{{[{(x-{x}_{i})}^{2}+{(y-{y}_{i})}^{2}+{(z-{z}_{i})}^{2}]}^{\frac{1}{2}}/{R}_{n}\}}\,,\\ Z(x,y,z)=\frac{{\sum }_{i=1}^{N}W\{{[{(x-{x}_{i})}^{2}+{(y-{y}_{i})}^{2}+{(z-{z}_{i})}^{2}]}^{\frac{1}{2}}/{R}_{n}\}Pol{y}_{i,z}(x,y,z)}{{\sum }_{i=1}^{N}W\{{[{(x-{x}_{i})}^{2}+{(y-{y}_{i})}^{2}+{(z-{z}_{i})}^{2}]}^{\frac{1}{2}}/{R}_{n}\}}\end{array}$$

where *R*_*n*_ represents the distance of point *p* from its (*n* − 1)^*th*^ nearest control point, and *W* is defined as follows in Equation (3) and Equation (4):

3 $$\begin{array}{rcl}W(R) & = & 1-3{R}^{2}+2{R}^{3},0\le R\le 1\\ W(R) & = & 0,R > 1\end{array}\}$$

4 $$R={[{(x-{x}_{i})}^{2}+{(y-{y}_{i})}^{2}+{(z-{z}_{i})}^{2}]}^{1/2}/{R}_{n}$$

The definition of *W* ensures that the polynomials $Pol{y}_{i,x}$,$\,Pol{y}_{i,y}$, and $Pol{y}_{i,z}$ will affect only those points whose distance from a control point $({x}_{i},{y}_{i},{z}_{i})$ in the reference image is lower than *R*_*n*_. Hence, it ensures local transformation. Moreover, as mentioned in Equation (5), the first derivative of the weight function with respect to *R* is 0 for the values *R* = 0 and *R* = 1. Therefore, the smoothness and continuity of the weighted sum is maintained for all image points even when the point is beyond the reach of the polynomial influence.

5 $${[\frac{dW}{dR}]}_{R=0}={[\frac{dW}{dR}]}_{R=1}=0$$

The transformation functions among all the image pairs of a cardiac cycle are calculated with respect to the reference frame. The calculated transformation function is further used for myocardial point tracking. The local deformation of the mesh is ensured by selecting *n* local control points to calculate transformation in local areas. The value of *n* is determined considering literature^28,31,32^ and small in-house analysis. A grid search over a selected range of values (10, 20, 30, …, 350) of *n* is performed. The smaller values of *n* increase tracking error, very large values may not be able to calculate local deformations of the image. Therefore, we choose *n* = 100 since it resulted in minimum myocardial tracking error.

#### Myocardial point trhacking and strain calculation

Myocardial point tracking and strain calculation steps are followed from continuum mechanics literature^37,38^. We consider the segmented LV myocardium points of the first frame as reference points, and track them using the estimated LWM transformation function. The tracking is performed in a forward tracking fashion from the first frame to the final frame of a cardiac cycle. The spatial location of the myocardial point $p(x,y,z)$ at time *t* is defined as ${[x,y,z]}^{T}={f}_{Deformation}(p,t)$. The spatial displacement vector at time t is calculated as a difference in spatial locations with respect to reference frame points $u(x,t)={[{u}_{x},{u}_{y},{u}_{z}]}^{T}$. The displacement gradient tensor $U(x,t)=\bigtriangledown u(x,t)$ is defined as follows (Equation (6)).

6 $$U=[\begin{array}{c}\frac{\partial {u}_{x}}{\partial x}\frac{\partial {u}_{x}}{\partial y}\frac{\partial {u}_{x}}{\partial z}\\ \frac{\partial {u}_{y}}{\partial x}\frac{\partial {u}_{y}}{\partial y}\frac{\partial {u}_{y}}{\partial z}\\ \frac{\partial {u}_{z}}{\partial x}\frac{\partial {u}_{z}}{\partial y}\frac{\partial {u}_{z}}{\partial z}\end{array}]$$

The displacement gradient tensor *U* is used to calculate deformation gradient tensor^37^ $F(x,t)$, which is mentioned in Equation (7). The computer code is adapted from MATLAB^39^ and updated with Lagranje strain definition.

7 $$F={(I-U)}^{-1}$$

As mentioned in Equation (8), the 3D Lagrange strain tensor^38^ $E(x,t)\,\,$can now be calculated using $F(x,t)$.

8 $$E=1/2({F}^{T}F-I)$$

After computing the global frame *X*, *Y*, *Z* we calculate longitudinal strain (E_L_), circumferential strain (*E*_*C*_), and radial strain (*E*_*R*_) using a local co-ordinate system. Global strain *E* is projected in a specific direction using the equation ${E}_{p}={p}^{T}\cdot E\cdot p$ where *p* represents a given direction (circumferential, radial, longitudinal). The longitudinal direction (*L*) is defined by drawing a line from apex to the mitral valve. The radial direction (*R*) is computed using a normal (*M*) at each node and longitudinal direction. $R=M-(M\cdot L)L$. Hence, the radial direction is at right angles to the epicardial border, and outwards. Circumferential direction (*C*) is calculated as a cross product of *L* and *R*. Hence, the direction of *C* is parallel to the epicardial border in the short-axis plane and counter-clockwise when observed from the base. We have adopted the definition of projecting strain in circumferential, longitudinal and radial direction from the benchmark framework^12^.

### Dataset and validation

The study involves a publicly available dataset of 3D tagged MRI volumes^12^. We used fifteen volumes, each one having a pixel size of 0.96 millimetre. The patient-specific imaging details such as a total number of slices, end-systolic frame number, and the total number of cardiac phases are different for each patient (see Table 1 for a complete report). Moreover, the patient-specific characteristics such as body surface area, age, and sex are also reported in Table 1.

**Table 1:** Dataset details.

ID	Patient	Modality	Age (year)	Sex	Body Surface Area (m^2^)	Total Cardiac Phases	End-Systolic Frame	Total Number of Slices
1	V1	3DTag	28	M	1.73	22	10	95
2	V2	3DTag	30	F	1.55	28	10	80
3	V4	3DTag	29	F	1.63	25	10	90
4	V5	3DTag	36	M	1.84	22	10	94
5	V6	3DTag	34	M	1.92	22	10	94
6	V7	3DTag	32	M	1.99	30	11	100
7	V8	3DTag	27	M	2.13	30	10	100
8	V9	3DTag	29	M	1.78	29	10	94
9	V10	3DTag	22	M	1.84	26	10	80
10	V11	3DTag	22	M	1.88	31	11	100
11	V12	3DTag	30	M	1.94	23	10	80
12	V13	3DTag	31	M	1.78	37	10	90
13	V14	3DTag	24	F	1.61	28	11	75
14	V15	3DTag	20	M	1.65	20	8	90
15	V16	3DTag	20	M	2.06	24	9	90

We validated the proposed method using four strategies: (i) calculating the tracking error of ground truth landmarks, (ii) visualizing myocardial points displacement, (iii) performing strain calculation, and (iv) analysing eigenvalue curve. The last frame of the cardiac cycle is referred to as a final frame in each patient. The validation results are reported in the Results and Validation section.

## Results and Validation

### Tracking of ground truth landmarks

The first validation strategy of tracking ground truth landmarks was performed with the manually tracked landmarks by two different observers. The landmark details were obtained from the dataset provider^12^, and summarized as follows. The total number of landmarks per observer is 12, which are distributed all over the myocardial wall. One landmark per each anterior, septal, posterior, and lateral wall at each level basal, apical, and mid-ventricular is placed. The inter-observer variability by comparing relative positions of the landmarks was computed to include the landmark for validation. If the final position and initial position of the landmark in each individual observer are relatively close, and the final position of the landmark is relatively close in both observers then the landmark is considered as the ground truth landmark. The position is referred to as relatively close if the distance is less than the 75^th^ percentile of all measured distances.

**Figure 5:**
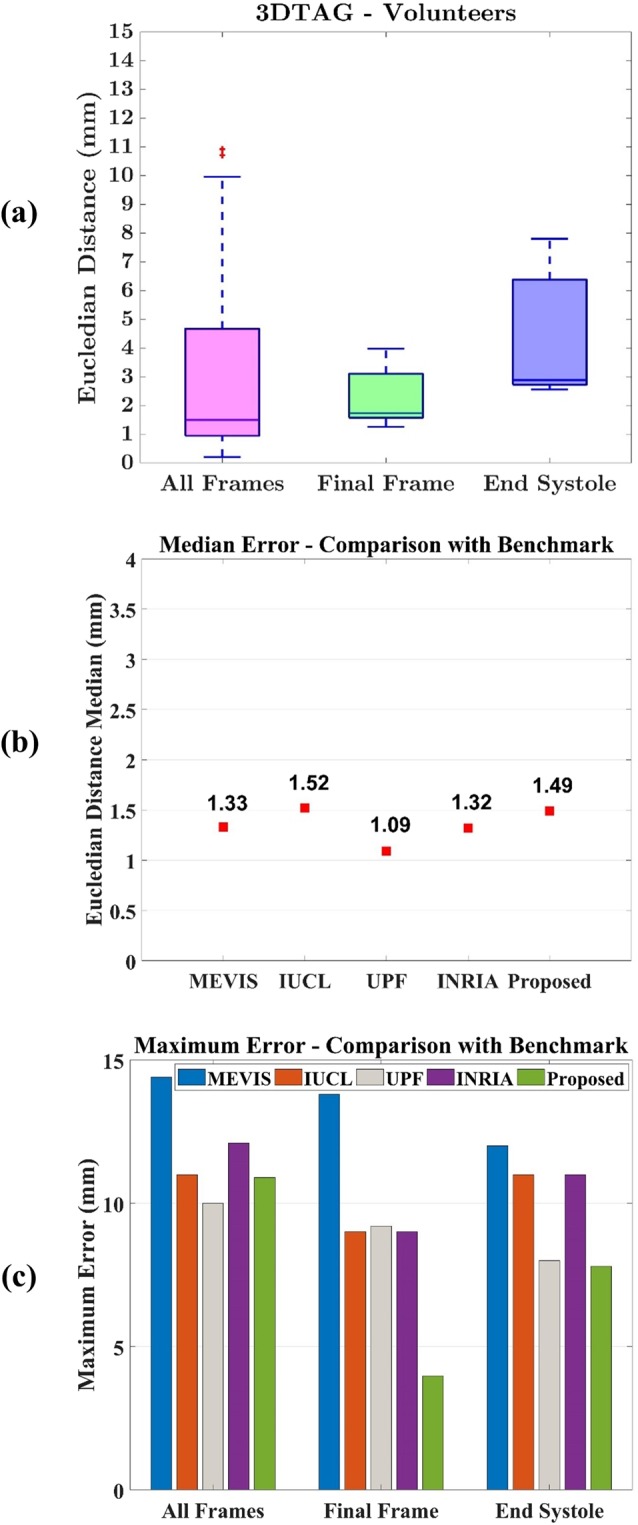
(**a**) Tracking error of the proposed method, **(b)** Comparison of median error with benchmark methods, **(c)** Comparison of maximum error with benchmark methods.

The landmarks are tracked over all the cardiac frames using 3D TAG patients dataset. As mentioned in Fig. 5(a), the tracking error is reported at all frames together, at end-systolic frames and at final frames. The median error using the proposed method at all frames is 1.49 mm, at final frames are 1.73 mm, and at end-systolic frames is 2.88 mm.

The errors are compared with benchmark methods MEVIS (Fraunhofer MEVIS, Bremen, Germany), IUCL (Imperial College London – University College London, UK), UPF (Universitat Pompeu Fabra, Barcelona, Spain), and INRIA (Inria-Asclepios project, France). As mentioned in Fig. 5(b), the median error using the same 3DTAG dataset are MEVIS = 1.33 mm, IUCL = 1.52 mm, UPF = 1.09 mm, INRIA = 1.32 mm, and HTM(proposed method) = 1.49 mm. The results show that the proposed method has a similar median error to one of the benchmark methods, and half a millimetre higher error compared to other benchmark method.

Moreover, as shown in Fig. 5(c), the maximum error (considering all the frames) is MEVIS=14.4 mm, IUCL=11 mm, UPF = 10 mm, INRIA = 12.1 mm, and the proposed method HTM = 10.9 mm. The HTM has reduced 24.3% maximum error compared to MEVIS, 0.9% compared to IUCL, −9% compraed to UPF, and 9.9% compared to INRIA. The highest maximum error is reported with MEVIS method, and the lowest is reported with UPF method. The proposed method has a higher error than lowest. However, as mentioned in Fig. 5(c), the proposed method has the lowest error at the end-systolic frame and final frame compared to other benchmark methods. At final frames, the highest error is reported by MEVIS (13.8 mm) and the lowest error is reported by the proposed method (HTM = 3.97 mm). At end-systolic frames, the highest error is reported by MEVIS (12 mm) and the lowest error is reported by the proposed method (HTM = 7.8 mm).

### Displacement of myocardial points

The second validation strategy is to observe displacement at the end-systolic frame and the final frame with respect to the reference frame. The end-systolic frame should have a higher displacement. The myocardium points of the segmented LV were tracked in all the frames of a cardiac cycle and the displacement is visualized for the end-systolic and final frame. The displacement of myocardial points was measured as the point-to-surface distance in millimetre. The point-to-surface distance is defined as the distance of a point from the end-diastolic surface.

**Figure 6:**
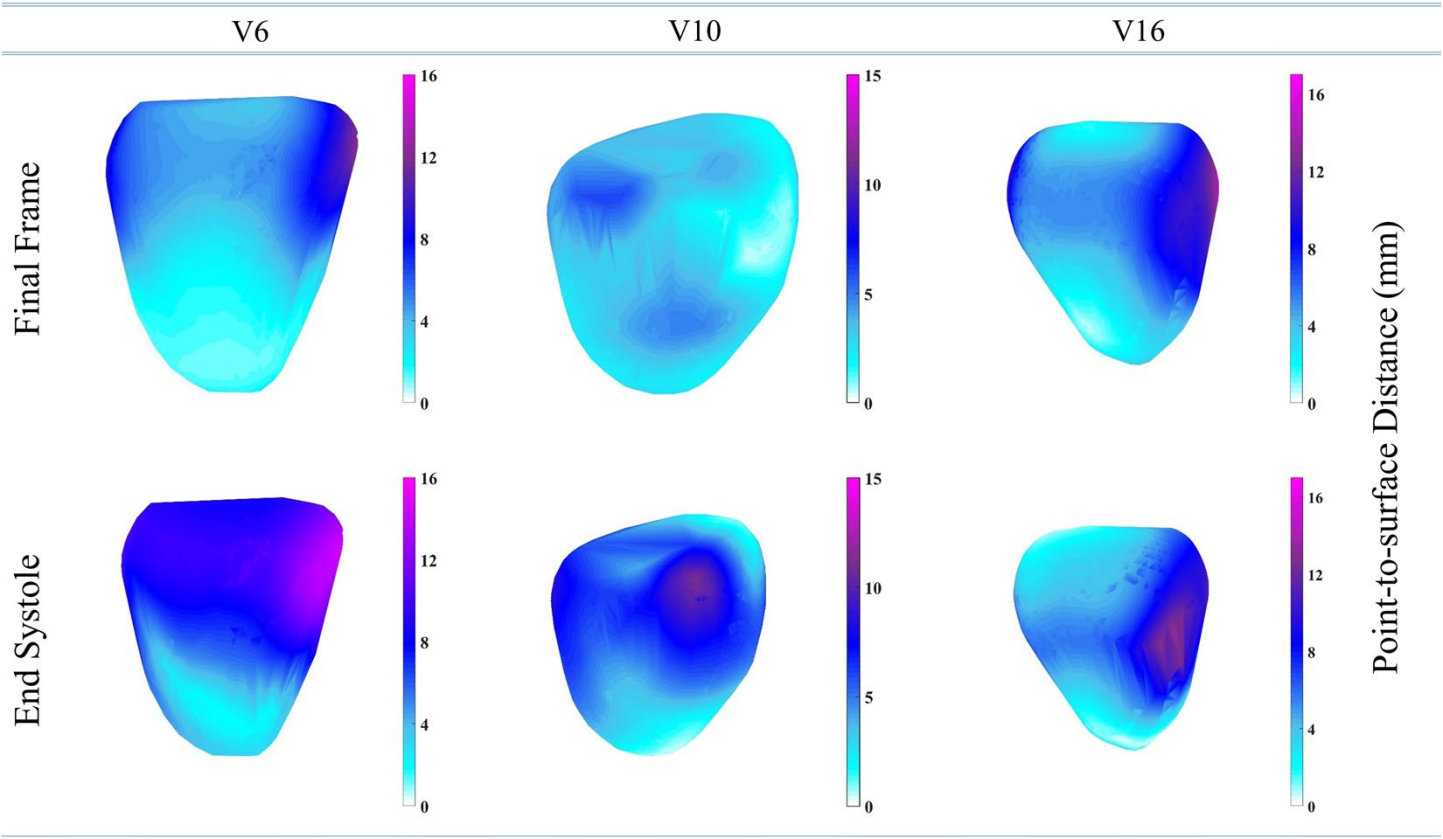
Displacement comparison at the end-systolic frame and final frame. The three patients (V6, V10, V16) with different left-ventricle walls are shown. Point-to-surface distance is a measure to estimate the distance of a point from the reference surface.

The end-systolic frame has average 8 mm to 18 mm of point-to-surface distance, and the final frame has 0 to 6 mm point-to-surface distance over the LV wall. We have divided the original dataset into three categories as per the quality of images: (i) average images, (ii) good images, (iii) excellent images. In Fig. 6, we have chosen an example from each category. As shown in Fig. 6, the displacement of myocardial points at end-systole is higher compared to the final frame. The end-systolic frame of patient V6 has a higher displacement in basal, mid-ventricular, and some of the apical area compared to the final frame. The patient V10 has a higher displacement in basal, mid-ventricular, apical, and some of the apex area compared to the final frame. The patient V16 has a higher displacement in the mid-ventricular area compared to the final frame. The similar point-to-surface distance is observed in the literature^12^.

### Strain analysis

The third validation strategy is the analysis of strain values. The myocardial strain for each frame was computed by estimating the displacement with respect to the end-diastolic frame. The strain tensor is computed as described in the Methods section. In order to validate the results, the strain values in all the cardiac frames are plotted. The peak strain values are compared with literature^40^. Moreover, the pattern of myocardial strain values in healthy patients is also analysed with physiological ground truth function of LV.

**Figure 7:**
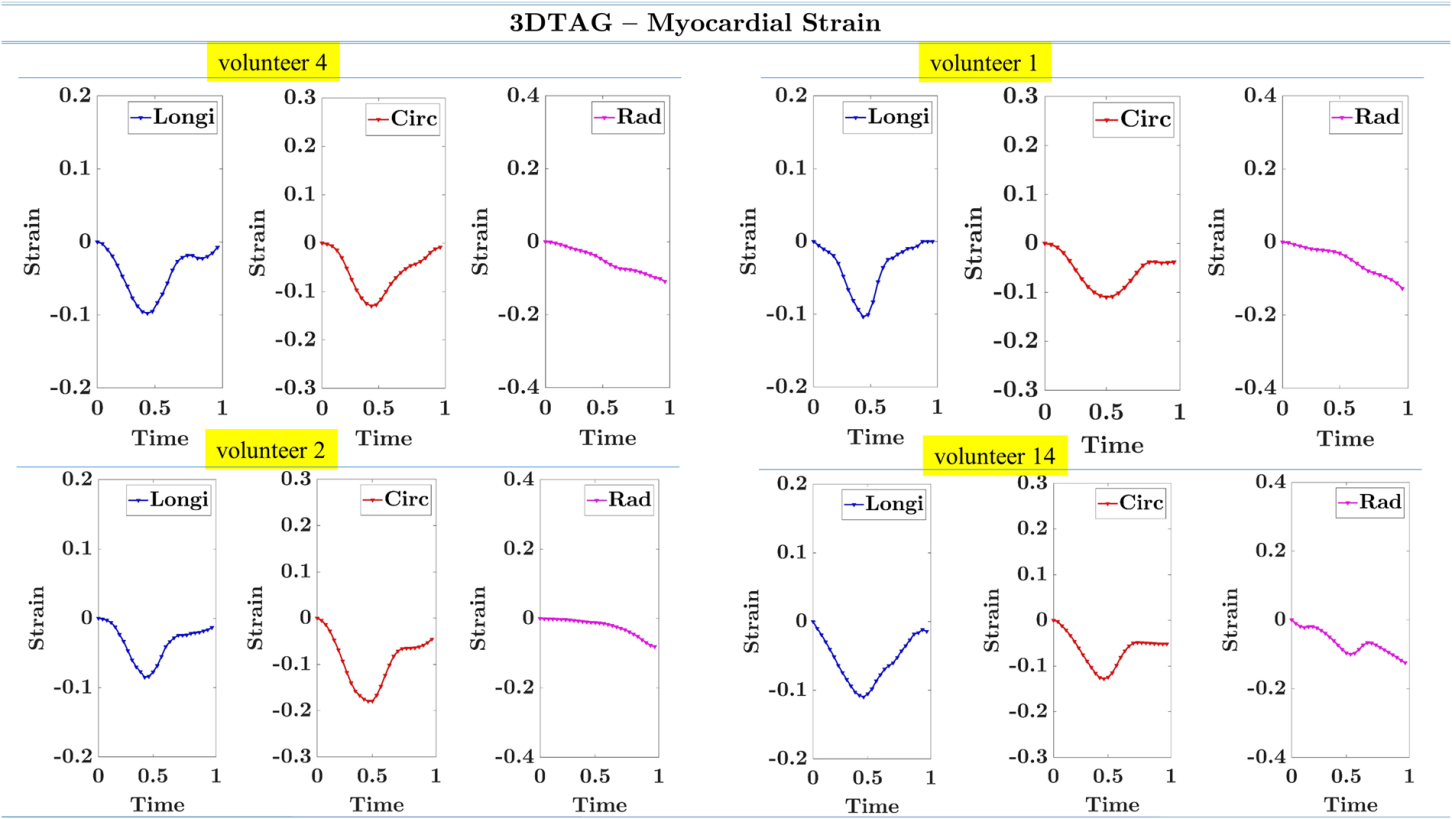
The longitudinal, circumferential, and radial strain plots using all LV points in patients V4, V1, V2, and V14. Longi: Longitudinal Strain, Circ: Circumferential Strain, Rad: Radial Strain.

Figure 7 shows the strain curves in longitudinal, circumferential, and radial directions using all LV points. Strain in four patients V4, V1, V2, and V14 is shown. Two patients have excellent quality images, one has good quality images, and one has average quality images. The longitudinal and circumferential strain have negative values. Moreover, the end-systolic frame has a peak value of strain. The strain is increased at the beginning of a cardiac cycle, and decreased during the latter part. The radial strain is reported almost zero. The similar observation is mentioned in the literature using the same dataset^12^.

### Eigenvalue analysis

**Figure 8:**
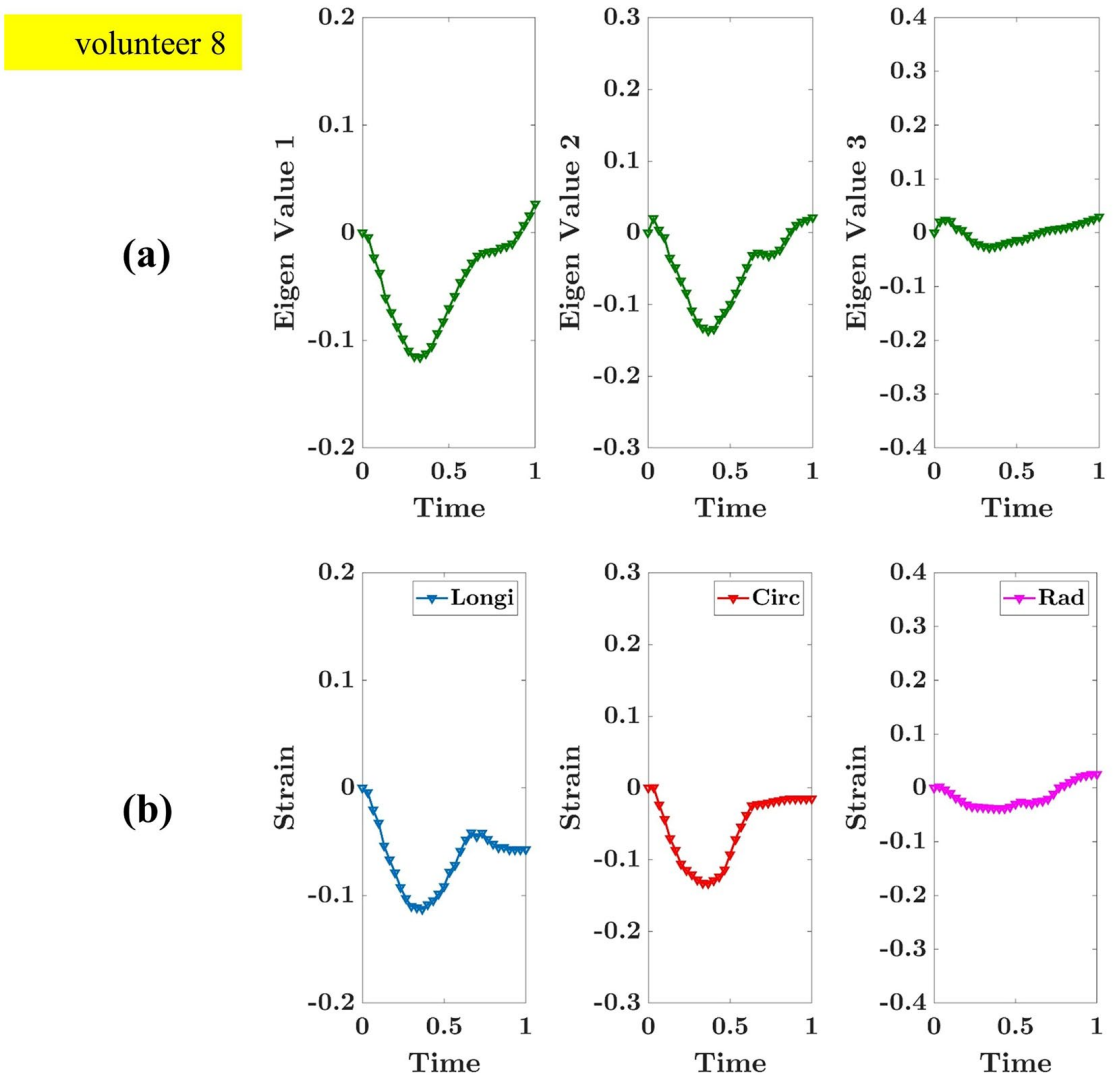
(**a**) Eigenvalue curve during a cardiac cycle in subject V8, **(b)** Strain value curve during a cardiac cycle in V8. Longi: Longitudinal Strain, Circ: Circumferential Strain, Rad: Radial Strain.

The fourth validation strategy is based on eigenvalue analysis. The strategy is adopted from a protocol in bio-mechanics literature^41–43^. The entire LV wall is considered as a finite collection of points, and, at each point, a Lagrange strain tensor is calculated (as mentioned in Equation 8). The eigenvalues of each strain tensor are calculated, and each strain tensor returned three eigenvalues. After that, threes sets of eigenvalues are prepared, and a median of each set is evaluated. This step provided us with a median eigenvalue in one frame of a cardiac cycle. A similar process is repeated for all the frames of a cardiac cycle to generate an eigenvalue curve during a cardiac cycle. The eigenvalue curve of subject V8, which has good quality images, is shown in Fig. 8. An additional analysis of longitudinal, circumferential and radial strain is also reported for the same subject.

As shown in Fig. 8(a), the eigenvalue curve has increased values at the beginning and decreased values in the latter part of the cardiac cycle. The peak eigenvalues and peak strain values are reported at the same end-systolic frame. Eigenvalue 1, eigenvalue 2, longitudinal strain and circumferential strain are reported with mostly negative values. Eigenvalue 3 and radial strain are reported with values closer to zero. The discussion of the results is reported in the Discussion section.

## Discussion

The article has contributed a 3D myocardial tracking and strain calculation methodology to reduce myocardial tracking error. The novel aspects of the article are as follows:

- Myocardium tracking can be performed with 1.49 mm median error and a very few outliers (numerically stable).
- Maximum myocardial tracking error is reduced up to 24% compared to the benchmark methods.
- Extension of the previously published 2D method^25^ for 3D, which can provide simultaneous 3D myocardial strain.
- To the best of authors’ knowledge, the introduction and adaptation of LWM function in 3D myocardial tracking pipeline.

The circumferential and longitudinal strain curves could provide insights into cardiac MRI-based diagnosis. As the 3D TAG MRI data which is used in the article is an emerging imaging technology for research, the standardization of 3D TAG MRI protocol is necessary before using it for real-world applications. In the future, the proposed methodology could potentially aid the diagnosis of myocardial infarction patients to locate akinetic segments and understand ventricular remodelling^2,44^, diagnosis of cardiac resynchronization therapy patients to locate dyskinetic segments^2,5^ valvular disease patients^2^. The strain values calculated from this method could be used in 3D finite element model to predict *in-vivo* myocardial properties^45^, and subsequently, estimate the ventricular wall stress for healthy^46,47^ and diseased patient^48^ for improved understanding of cardiac biomechanics. The 3D LWM function is written in C/C++ for reusability, which is an extension of the MATLAB function and source code for 2D images^49^. Then LWM is used with MATLAB code for the remaining methodology. The discussion of results is mentioned in the further sub-sections. The results, validation, and discussion are using healthy patients. However, a similar approach can be extended for diseased patients.

### Tracking of ground truth landmarks

As mentioned in Fig. 5, the proposed method has a similar median error to one of the benchmark methods, and half a millimetre higher error compared to other benchmark methods. However, the tracking error at all frames, final frames, and end-systolic frames do not have large outliers. As mentioned in Section 2.1, the maximum error is up to 24% reduced in the proposed method compared to the benchmark methods. The reason is the technical advantages of LWM transformation function. LWM functions do not require to solve a very large system of linear equations, and local polynomials can be efficiently solved with a small system of equations, which helps to avoid ill-conditioned polynomials. As a result, tracking errors are more consistent and do not have large outliers. Moreover, LWM function can adapt the local geometrical shape and different density of points, and as a result, landmark tracking does not require a specific type of coordinate systems such as mentioned in the literature^30^ which also helps to avoid ill-conditioned polynomials and large error outliers. In addition, a large number of control points with relatively uniform distance is available, and the averaging process of the transformation function may help to smooth any noise in corresponding landmarks as suggested in the literature^28^, which may help to improve tracking when tag points of MRI suffer from fading issues. The time complexity of 3D LWM transformation for a *U* × *U* × *U* size 3D image is *O*(*NTU*^3^), where *N* refers to the number of linear equations while solving a system of equations (same as local control points *n*) and *T* refers to the number of co-efficients in a polynomial. The proposed method has used second order polynomials with ten coefficients.

### Displacement of myocardial points

As mentioned in the dataset section, all the scans are from healthy patients. Therefore, the LV muscles should have a higher contraction at end-systolic frame compared to other cardiac frames. Our results support the fact that the end-systolic frame of a healthy patient should have a higher displacement compared to the displacement at the final frame, hence follow the expected physiological pattern. The values of point-to-surface distance are smooth over LV which shows that the LWM function provides a smooth transformation (mathematical characteristic of smoothness is mentioned in equation (5)).

### Strain analysis

As per the physiology of the healthy human heart, LV contracts in longitudinal and circumferential directions and expands in the radial direction during systole. Therefore, circumferential and longitudinal strain values are expected to be negative with an increased strain in the beginning and reduced strain in the later part of a cardiac cycle. This fact is highlighted in our results of Fig. 7, which follows the expected physiological pattern. However, the average peak strain is expected to be −20% in the circumferential direction, −16% in the longitudinal direction, and +45% in the radial direction^40^. The reported peak strain is reduced compared to the literature^40^, which shows that the methodology and dataset need further advancements before using it for real-world applications. It is indicated in the literature^12^ that the 3D TAG MRI protocols are under research, therefore, the derived strain values using different methodology are reduced. However, this may provide insights to further improve 3D imaging sequences which ultimately could improve dataset and strain calculation.

### Eigenvalue analysis

It is expected that the eigenvalue curve, similar to the strain curve, follow a physiological pattern of LV contraction and expansion. The results of eigenvalue 1 and eigenvalue 2 are decreased in the beginning and increased in the later part of the cardiac cycle which is expected. In addition, peak eigenvalues and strain values are reported at the end-systolic frame, which is expected. However, our observation shows that the eigenvalue which is less than zero is corresponding to the LV contraction. In literature^41^, it is observed that the eigenvalue less than one corresponds to the LV contraction. This difference is due to the different definitions of the strain tensor in both literature^12,41^. It is observed that both researchers have reported a similar shape in the eigenvalue curve. It is observed that the eigenvalues 3 are closer to zero and the range of eigenvalues (between maximum and minimum) is reduced in our results compared to the literature^41^. The potential reason is the use of a different subject and different imaging modalities as they have used echocardiography images whereas our model is based on 3D TAG MRI. This observation could be insightful to further improve 3D TAG MRI acquisition protocol. A similar observation with strain values is made by the researchers who worked on the same dataset^12^.

In eigenvalue analysis, the derived three eigenvalues correspond to three principal strains, and a corresponding eigenvector designates a principal direction of associated with a principal strain. Principal strains (maximum and minimum normal strain), are diagonal elements of a strain tensor when off-diagonal (shear strain) is zero. Therefore, eigenvalue analysis provides principal strain (maximum and minimum normal strains) when shear strain is zero. However, longitudinal, circumferential, and radial strain are easier to interpret and communicate with clinical experts as they are common in clinical practise although they do not convey maximum/minimum normal strain and includes shear strain. Therefore, considering a technical and a clinical scenario, both analyses are crucial.

### Limitations and future work

The results are derived using a benchmarking dataset of 3D + time MRI to evaluate the proposed methodology and technical contributions. It should be noted that our results can not be directly used as a clinical measure. Before concluding any generalised clinically interpretable results, the proposed methodology has to be tested with a large number of data samples. We consider collecting a large number of 3D + time data as future work. The proposed method has difficulty in capturing radial strain, which is reported by other researchers as well^12^. As a possible solution and future work of this article, we are considering to merge different modalities such as 3D steady-state free precision to improve the calculation of radial strain using the proposed method.

It should be noted that our contribution is not limited to only cardiac 3D MRI analysis. The transformation function, which is extended in this article for 3D, can be used with other application areas where 3D imaging has already established standards. For example, finding defects in industrially manufactured parts could be possible with 3D computed tomography image analysis^50^. Such image analysis of registration-based methodology could use the contributed 3D transformation function. Similarly, the exploration of natural resources could be possible with landmark-based geo-spatial image registration^32,34^. Such methodologies can utilize the contributed 3D transformation function. Moreover, 3D speckle tracking methods^17^ with 3D ultrasound images can utilize the contributed transformation function. Such applications are considered as future applications of this article.

## Conclusion

In this article, we proposed a 3D myocardial tracking and strain calculation method. The novel aspect of this method is the adaptation of LWM function for 3D myocardial tracking problem. The function is extended for 3D, and adapted for 3D myocardial tracking and strain calculation. The proposed method can perform myocardial tracking without using a specific type of coordinate system and elaborated spline process which can help to avoid ill-conditioned polynomials and large errors. As a result, the myocardium tracking is numerically stable with 1.49 mm median error, and the maximum error of tracking is reduced up to 24% compared to benchmark methods. The protocol of eigenvalues and derived strain values can provide insights to the researchers working on 3D TAG MRI imaging sequences. Moreover, the article could be insightful for the 3D image analysis in manufacturing, and geoinformatics. The contributed computer code of the transformation function can be directly utilized for landmark-based image registration techniques of 3D images. In the future, after the development and standardization of 3D TAG MRI, the article could be useful for real-world applications of cardiac MRI-based diagnosis.

## Data Availability

The computer code of the method is available from the corresponding author on a reasonable request. The original MRI scans with user guidelines can be accessible as mentioned in the literature^12^.
